# Community First Theory: How Collective Organization Generates Individual Diversity

**DOI:** 10.3390/e28050523

**Published:** 2026-05-05

**Authors:** Takashi Ikegami, Hiroki Kojima, Akiko Kashiwagi

**Affiliations:** 1 Graduate School of Arts and Sciences, The University of Tokyo, 3-8-1 Komaba, Meguro-ku, Tokyo 153-8902, Japan; 2 Alternative Machine Inc., Tokyo 150-0001, Japan; 3 Faculty of Agriculture and Life Science, Hirosaki University, Bunkyo-cho 3, Hirosaki 030-8561, Aomori, Japan; 4 The United Graduate School of Agricultural Science, Iwate University, Ueda-3, Morioka 020-8550, Iwate, Japan

**Keywords:** community, NTIC, *Tetrahymena*, individual tracking

## Abstract

Collective systems often exhibit emergent behaviors that cannot be reduced to the properties of individual components. A central question is whether individuality itself is a precondition for collective organization, or whether it arises *from* it. Here we develop and empirically test *Community First Theory*, which proposes that collective organization is the generative substrate from which individual dynamical identity emerges. To operationalize this claim, we introduce non-trivial information closure (NTIC), which quantifies whether an individual’s temporal predictability is self-determined or distributed across collective relations. Using high-resolution tracking of complete *Tetrahymena* populations across four generations, we show that information closure emerges transiently in the middle phase of the cell cycle, flanked by strong collective coupling. Cells in the information-closed regime show significantly greater divergence from parental phenotypes, demonstrating that community organization actively generates behavioral diversity. These results provide initial empirical support for Community First Theory in a single-model system and suggest that NTIC offers a substrate-independent tool for locating agency transitions in collective systems.

## 1. Introduction

This paper examines how collective organization shapes individual identity in clonal populations of *Tetrahymena thermophila*, a ciliated protozoan that undergoes synchronized binary fission. Using complete single-cell tracking across four successive fission generations, we find that autonomy, informational diversity, and life-history individuality do not precede the formation of a community; rather, they emerge from it. We call this principle the *Community First Theory*: collective dynamics serve as the generative substrate from which individuality is progressively differentiated [1,2].

The present paper concentrates on *Tetrahymena* as a controlled model system; parallel investigations of honeybee societies [3], ant colonies [4], and multi-agent large-language-model environments [5] are reported separately. It has long been recognized that genetically identical cells can exhibit substantial phenotypic heterogeneity through stochastic gene expression [6,7], and that such noise-driven diversity can serve adaptive functions including bet-hedging [8,9] and division of labor [10]. Raj and van Oudenaarden [11] framed this as the interplay of Nature, Nurture, and Chance; the present work adds a fourth factor—*community*—arguing that collective interaction actively structures phenotypic diversity beyond what intrinsic noise alone can produce. Prior quantitative studies of *Tetrahymena* behavior have established rich single-cell phenotypic diversity [12] and homeorhetic community dynamics [13], and single-cell RNA sequencing of the same organism has revealed distinct transcriptional subpopulations within clonal cultures [14]. Those experiments operated at low cell density where inter-individual interactions are minimal; our confined microchamber setup places all cells in a strongly interacting regime, making community formation rather than individual phenotyping the primary object of study.

To operationalize this hypothesis we use *non-trivial information closure* (NTIC) [15], defined as

(1) $$\mathrm{NTIC}\;=\;I\left(X_{t+1};\;X_{t}\right)\;-\;I\left(X_{t+1};\;X_{t}|Z_{t}\right),$$

where I(·;·) denotes mutual information, I(·;·∣·) denotes conditional mutual information, $X_{t}$ is the kinetic energy of a focal cell, and $Z_{t}$ is the mean kinetic energy of its contemporaries [1,16]. In [15], the “environment” is external to the system of interest; here, $Z_{t}$ is the community of conspecifics—other cells of the same kind. This difference is not incidental but central to Community First Theory: the relevant environment from which individuality emerges is the collective itself. A positive NTIC indicates that the cell’s own past predicts its future beyond what community context can explain, whereas NTIC near zero signals that self-prediction and community-prediction operate on independent (orthogonal) channels of the future state.

A key concept underlying our framework is *information closure*: the condition that a system’s future dynamics can be predicted from its own past without requiring additional explanatory variables [16]. In collective biological settings, however, closure does not imply isolation. An individual component may remain statistically embedded in a correlated community while its temporal predictability becomes effectively self-determined. This *situated autonomy*—agency emerging *within* collective organization rather than outside it—is precisely what NTIC is designed to detect [17]. When NTIC≈0 yet coupling to the community is non-negligible ($I(X_{t};\;Z_{t})>0$), the cell is information-closed: it has become a coherent individual without having severed its community ties.

NTIC connects naturally to partial information decomposition (PID) [18,19,20], which decomposes the total predictive information about $X_{t+1}$ carried jointly by $X_{t}$ and $Z_{t}$ into four atoms: unique contributions from each source, redundancy shared by both, and synergy accessible only through their combination. NTIC approximates the difference

(2) NTIC = Redundancy−Synergy.

Positive NTIC (redundancy exceeds synergy) identifies self-sustaining individuals: the cell’s past and community context carry overlapping predictive information, a signature of stable, role-bearing agents. NTIC near zero with non-zero coupling signals that redundancy and synergy are balanced—the hallmark of situated autonomy. Crucially, high synergy (NTIC<0) should not be interpreted as a more advanced state; within Community First Theory, synergy-dominated regimes correspond to a *pre-specialization* phase in which individual roles have not yet stabilized and predictive structure is accessible only through relational combinations. Computing synergy therefore allows us to distinguish cells that are genuinely self-determining from those whose predictability arises from transient contextual dependence.

Our main empirical findings are threefold. First, NTIC tends to be largest early in a cell’s life and declines over the life history, indicating that young cells bear a strong individual-level predictive structure that is gradually shared with the community. Second, fidelity of parent–daughter kinetic-energy distributions was assessed in the quasi-stationary middle phase only—the early phase immediately after division (when cells move slowly) and the late phase immediately before the next division (when cells again slow) are excluded as confounded periods unsuitable for inheritance estimation. Within this middle phase, coupled-regime cells show significantly higher distributional fidelity than information-closed cells (Mann–Whitney U = 304, $p_{\mathrm{corr}}\;=\;0.041$, r = 0.37), demonstrating that community coupling actively generates, rather than suppresses, behavioral diversity. Third, the connection between NTIC and partial information decomposition reveals that information-closed cells balance redundancy and synergy in a way consistent with *situated autonomy*: remaining embedded in community context while maintaining effective self-prediction.

In the following sections, we formalize this framework and apply it to fully tracked multicellular-like dynamics in *Tetrahymena*.

## 2. Materials and Methods

### Experimental Setup and Data Acquisition

**Figure 1:**
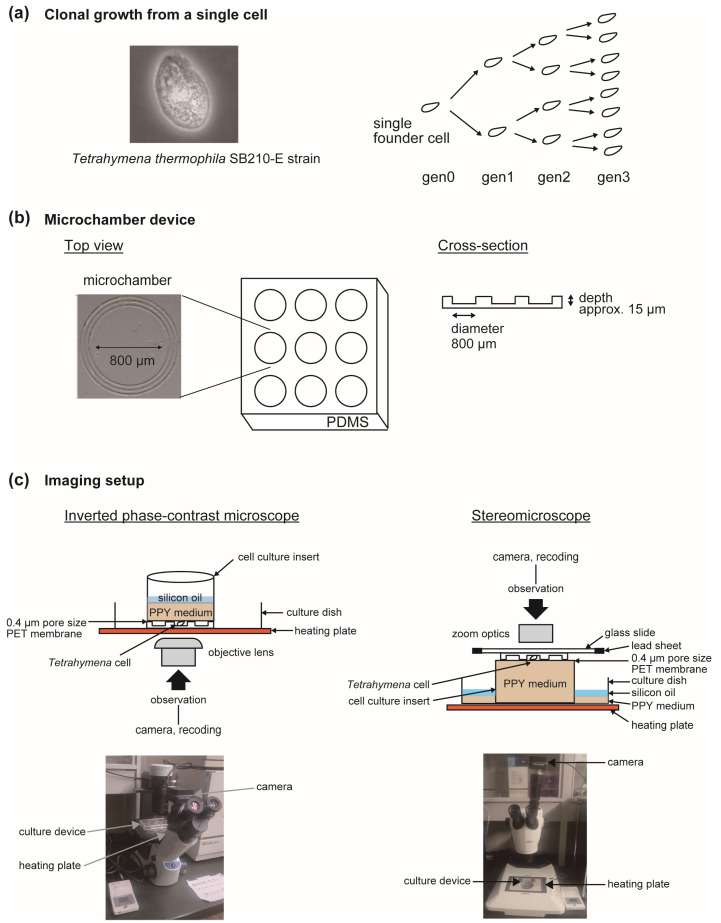
Experimental system for long-term tracking of single-cell growth and motion. (**a**) *Tetrahymena thermophila* and schematic of binary fission from one to eight cells. (**b**) PDMS microchamber device. Each cylindrical chamber was approximately 800 μm in diameter and 15 μm in depth. A sheet-type PDMS device containing nine chambers arranged in a 3 × 3 array was fabricated for experiments. (**c**) Imaging configurations used for continuous tracking. In the inverted phase-contrast setup, chambers were covered with a cell culture insert equipped with a 0.4 μm pore-size PET membrane. Culture medium and an overlying silicone oil layer were applied to permit oxygen diffusion while suppressing evaporation. In the stereomicroscopic configuration, the chamber device was inverted onto a membrane insert and mechanically stabilized using a weighted glass slide. In both cases, the device was maintained at 35 °C on a temperature-controlled stage during recording.

We tracked the collective swimming dynamics of *Tetrahymena thermophila* in a confined microenvironment enabling long-term observation across successive binary fissions (Figure 1a). The *T. thermophila* SB210-E strain (Tetrahymena Stock Center, Cornell University) was cultured in PPY medium (1% proteose peptone, 0.15% yeast extract, 0.01 mM FeCl_3_) [21].

Cells were loaded into a sheet-type PDMS device containing nine cylindrical microchambers (800 μm diameter, approximately 15 μm depth) (Figure 1b), allowing continuous tracking from a single founder cell to the eight-cell stage (four generations). Chambers initially containing a single cell were selected for recording.

Two complementary imaging configurations were used (Figure 1c). For inverted phase-contrast microscopy (datasets 190308 and 190316), chambers were sealed with a PET membrane culture insert (0.4 μm pore size), supplied with PPY medium and overlaid with silicone oil to suppress evaporation while permitting oxygen diffusion. For stereomicroscopy (datasets 200617, 201002, 201003, 210818, and 210824), the device was inverted onto a membrane insert and mechanically stabilized with a weighted glass slide. In all experiments, recordings were performed at 35 °C on a temperature-controlled stage. Details of the experimental settings are provided in Appendix B.

Time-lapse videos were acquired at a temporal resolution of Δt = 100ms per frame, yielding complete trajectories across ∼14 h of observation (7 series, total N = 105 tracked cells). Cell trajectories were extracted using the Baxter Algorithms [22,23]. From these trajectories, instantaneous velocity v(t) and kinetic-energy proxy $KE\left(t\right)\;=\;\frac{1}{2}v{\left(t\right)}^{2}$ were computed for subsequent information-theoretic analyses. All mutual-information and conditional-mutual-information quantities were estimated with the *k*-nearest-neighbour (KNN) method [24,25] on log-transformed KE values. Inheritance fidelity was quantified by the Jensen–Shannon divergence [26] between parent and daughter KE distributions. Statistical significance of NTIC and MI(X;C) was assessed with a surrogate-data procedure [27] (50 permutations of $Z_{t}$ per cell; see Appendix A). The KNN parameter was set to k = 8 throughout. Mean NTIC values were stable across k∈{3, 5, 8, 10, 12} (middle-phase mean 0.029–0.035 bits), and per-cell NTIC estimates were highly correlated between neighbouring *k* values (Pearson r = 0.95–0.98 for k∈{8, 10, 12}; r = 0.91 for k = 5 vs. k = 8), confirming that k = 8 lies within a stable plateau and that the information-closure signal is not an artifact of the estimator bandwidth.

## 3. Results and Interpretation

### 3.1. Kinetic-Energy Dynamics Across Generations

**Figure 2:**
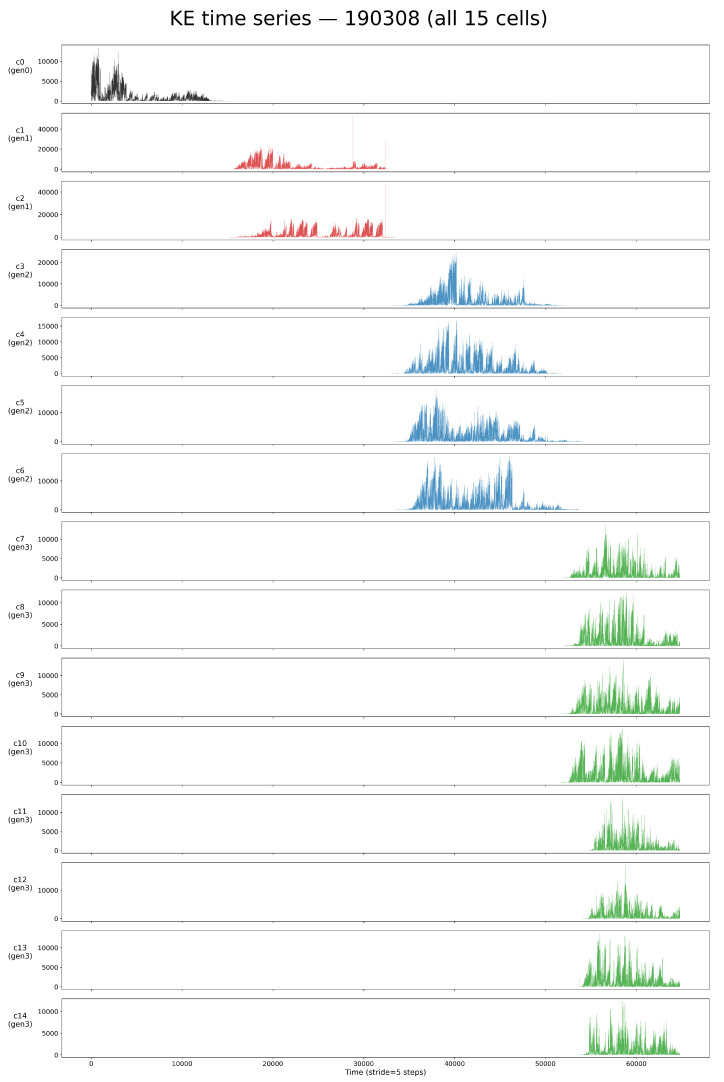
Kinetic-energy (KE) time series of all 15 cells in the 190308 genealogical tree. Each row shows the KE time series over the lifetime of a single cell, ordered by generation: gen0 (c0, black), gen1 (c1–c2, red), gen2 (c3–c6, blue), and gen3 (c7–c14, green). The total observation period is approximately 10 h. KE fluctuations reflect individual motility dynamics and form the basis for subsequent information-theoretic analyses.

We first examined the kinetic-energy (KE) time series obtained from long-term tracking of complete *Tetrahymena* lineages. Figure 2 shows a representative example (series 190308), where all cells from a single founder through to the eight-cell stage were continuously recorded. Each cell exhibits structured fluctuations in motility across successive fission events.

### 3.2. Distributional Phenotypes and Inheritance Variability

**Figure 3:**
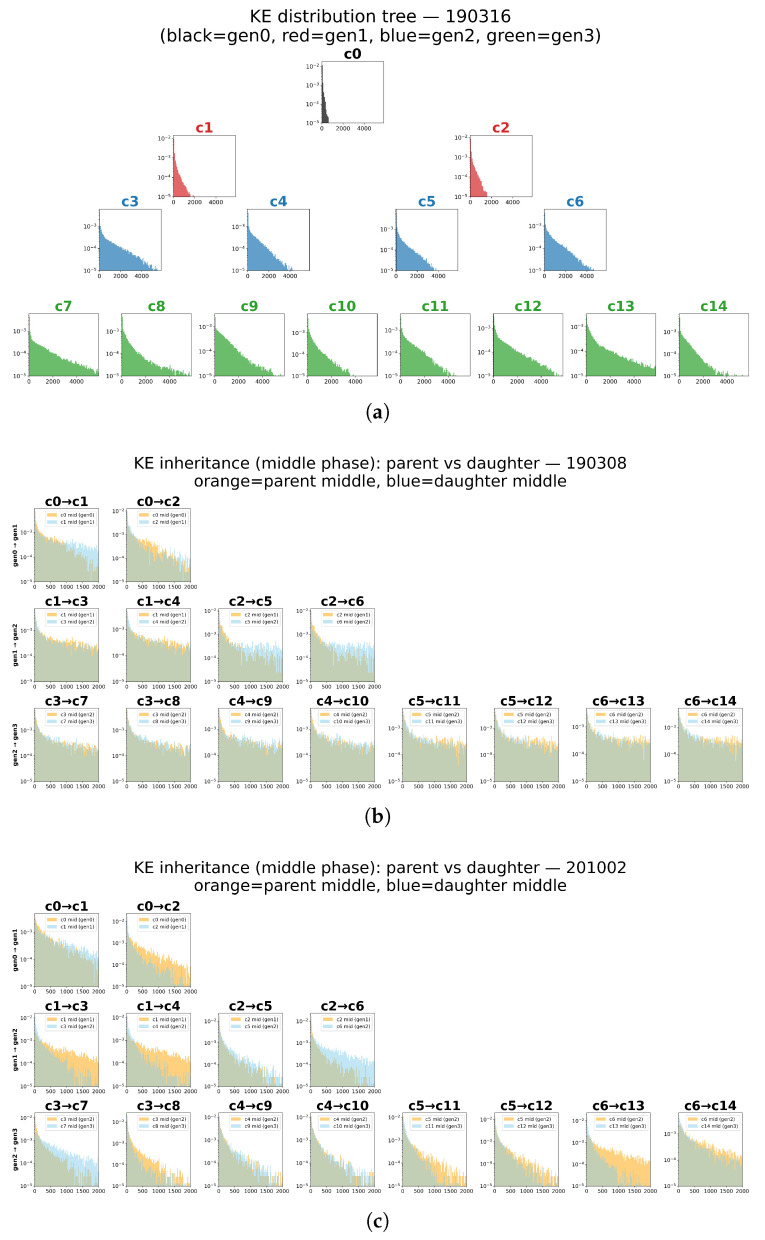
Inheritance of KE distribution shapes across generations. (**a**) Example lineage (190316). (**b**) Steep-decay distribution type (190308). (**c**) Heavy-tailed distribution type (201002). Overlaps between parent (orange) and daughter (blue) distributions illustrate heterogeneous inheritance fidelity across divisions. For consistency, distributions were estimated from the central 25 min window (3000 KE steps at stride 5, corresponding to 1500 s) centered on each cell’s midpoint between birth and subsequent division.

To characterize inter-generational changes in motility patterns, we compared KE distribution shapes across parent–daughter pairs (Figure 3). Two broad phenotypes were observed across the seven series: (i) a steep-decay type, with KE concentrated near zero and rapidly vanishing tails, and (ii) a broader heavy-tailed type extending to high-KE excursions. Importantly, inheritance was not uniform: within the same generation, sibling daughters could either preserve the parental distribution closely or diverge strongly, indicating that inheritance loss is often a cell-specific transition rather than a global lineage-wide shift.

### 3.3. Quantifying Inheritance Fidelity with Jensen–Shannon Divergence

To quantify inheritance fidelity systematically, we computed the Jensen–Shannon divergence (JSD) between parent and daughter KE distributions across all lineage series. At the same time, we asked how motility patterns evolve over generations: whether cells converge to a common motility attractor, or whether distinct attractors emerge through interactions among siblings within the developing collective. To address these questions, we extended the JSD analysis to all individuals in the population, comparing KE distributional shapes across cells.

**Figure 4:**
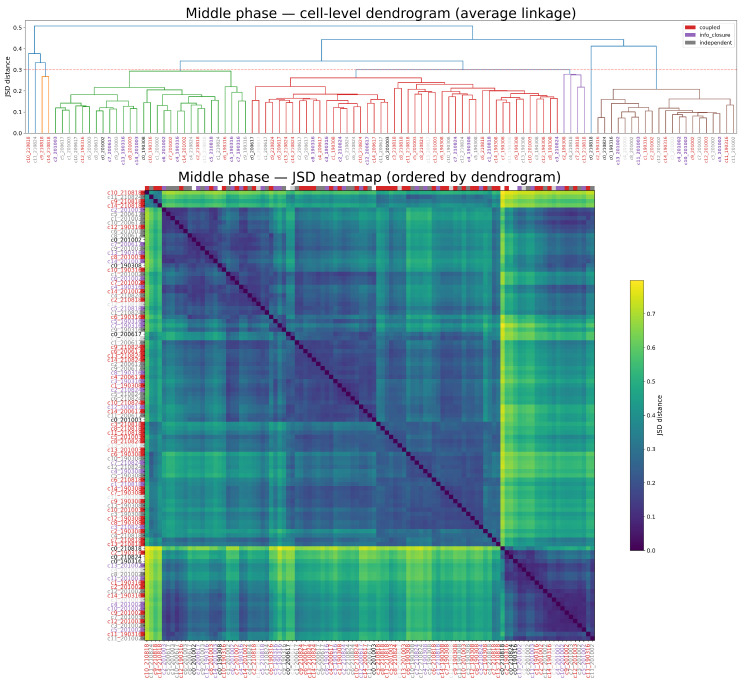
Pairwise Jensen–Shannon divergence (JSD) matrix for all 105 cells (15 cells × 7 series, generations 0–3), computed from middle-phase KE distributions. Each entry represents the JSD between a pair of cells; darker colours indicate more similar distributions and lighter colours greater divergence. Rows and columns are reordered by hierarchical clustering, revealing groups of phenotypically similar cells both within and across series. When the dendrogram is cut at the dashed line at y = 0.3, cells belonging to the same cluster are assigned the same color.

For consistency, distributions were estimated from the central 25 min window (3000 KE steps at stride 5, corresponding to 1500 s) centered on each cell’s midpoint between birth and subsequent division. Figure 4 reveals distinct inheritance modes: some lineages show consistently low divergence across generations (stable inheritors), whereas others exhibit localized inheritance “crashes” either early or mid-lineage. These results establish that motility attractors can be transmitted with high fidelity in some divisions, but can also undergo abrupt redistribution in others.

### 3.4. Information-Theoretic Quantification of Coupling and Closure

We next asked whether inheritance patterns relate to the distribution of predictive structure between individual histories and collective context. For cell *k*, we defined a sibling mean-field variable,

$$C_{k}\;=\;\frac{1}{N-1}\sum_{j\neq k}x_{j},$$

where *N* is the number of cells in the chamber and $x_{j}$ is the KE of cell *j*, and quantified non-trivial information closure (Equation (1)) where *X* denotes the KE state and $X^{\prime }$ its next-time state.

**Figure 5:**
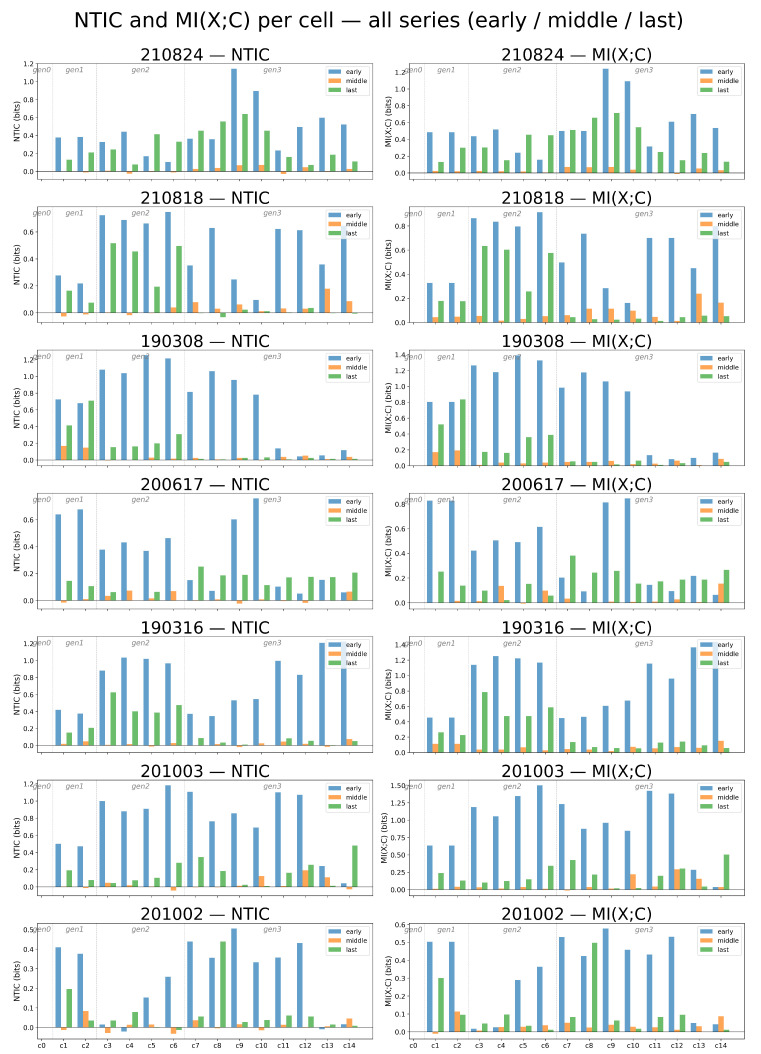
Per-cell NTIC and sibling coupling across seven complete *Tetrahymena* lineage series. (**Left**) panels show the magnitude of non-trivial information closure (NTIC) for each individual cell, while (**Right**) panels report the mutual information between the focal cell state and the sibling context, MI(X;C) (related to transfer entropy by MI(X;C) = TE(C→X)+NTIC). Bars are grouped by developmental phase (early, middle, and last) and separated by generation (1→2→4→8 cells). At the third generation (eight-cell stage), cells exhibit clear informational differentiation, allowing classification into coupled, information-closed, synergistic, and independent regimes based on the significance of MI(X;C) and the sign/magnitude of NTIC.

Cells were classified into three regimes (Figure 5): (i) *coupled* (I(X;C)>0, NTIC>0), (ii) *information-closed* (I(X;C)>0, NTIC≈0), and (iii) *independent* (I(X;C)≈0).

### 3.5. Phase-Wise Redistribution of Predictive Structure

Applying the classification scheme across the three life-history phases reveals a clear developmental trajectory.
**Phase****Coupled****Info Closure****Independent**Early97%1%2%Middle38%27%28%Last75%13%10%

Nearly all cells begin strongly coupled in the early phase. Information closure peaks in the middle phase, reflecting the transient emergence of *situated autonomy* within a correlated collective context. Coupling partially re-emerges prior to the next division (last phase), suggesting a non-monotonic reorganization of predictive structure across the cell cycle.

Categories are assigned using per-cell surrogate thresholds (50 permutations of $Z_{t}$ with $X_{t}$ held fixed).
MI (X;C) **Significant?****NTIC > Upper****NTIC < Lower****Category**yesyes–coupledyesnonoinformation closureyesnoyessynergistic ^∗^nononoindependentnoyes (either)anomalous ^†^

Upper and lower NTIC thresholds are the 95th and 5th percentiles of each cell’s surrogate distribution. MI(X;C) significance is assessed against the 95th percentile of the surrogate MI distribution. ^∗^ Exactly one cell met this criterion (experiment 201002, cell 6, generation 2, middle phase; NTIC =−0.039 bits), consistent with the expected 5% false-positive rate (1/98 = 1.0%); we treat this as a type-I error rather than a genuine synergistic regime. ^†^ Cells with non-significant MI(X;C) but NTIC outside the surrogate bounds; attributed to estimation noise and excluded from subsequent analyses (n = 7 in the middle phase, 7.1%).

The classification proceeds as follows. First, MI(X;C) is tested: is the real value above that cell’s 95th percentile of the surrogate $MI(X;\;C_{\mathrm{shuffled}})$ distribution? If yes, the cell genuinely shares information with siblings (p<0.05). Second, NTIC is tested: is the real NTIC above that cell’s 95th percentile of the surrogate ${\mathrm{NTIC}}_{\mathrm{shuffled}}$ distribution? If yes, the shared information genuinely contributes to self-prediction. Information closure is assigned when MI(X;C) is significant but NTIC is not: the cell is correlated with siblings, yet this correlation does not help predict the cell’s future beyond what one would get with a random *C*.

Per-cell thresholds are used rather than a single pooled threshold because different cells have different noise levels: a cell with noisier KE will have larger random fluctuations in MI estimates and therefore needs a higher threshold. In the present data, NTIC thresholds ranged from 0.022 to 0.048; a single pooled threshold would be too lenient for some cells and too strict for others.

### 3.6. Robustness Under Temporal Coarse-Graining

**Figure 6:**
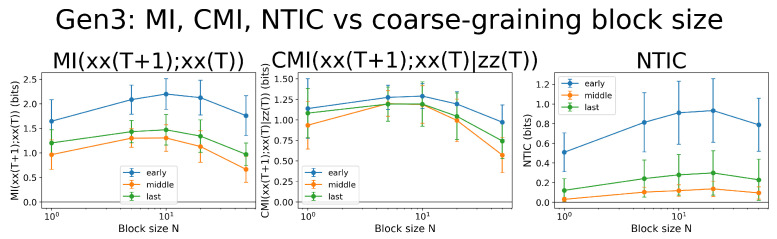
Robustness of information-theoretic quantities under temporal coarse-graining. KE time series were averaged over non-overlapping blocks of size N = 1, 5, 10, 20, 50, and three quantities were computed for every cell: $MI(X^{\prime };\;X)$ (**left**), $CMI(X^{\prime };\;X|Z)$ (**centre**), and NTIC (**right**). For block sizes N ≤ 10, middle-phase values exhibit the same trend, confirming that the observed informational structure is not an artifact of the chosen temporal resolution.

We further tested the scale dependence of NTIC by temporally coarse-graining KE time series into non-overlapping blocks of size *N* (Figure 6). While both mutual information and conditional mutual information varied with coarse-graining scale, the middle-phase NTIC remained close to zero across all block sizes examined (Table 1), confirming that information closure is not an artifact of the chosen temporal resolution but a robust feature of the middle-phase predictive structure.

### 3.7. Inheritance Fidelity Depends on Informational Regime

To test whether informational regime predicts inheritance fidelity, we computed $\sqrt{\mathrm{JSD}}$ between parent and daughter KE distributions in the middle phase only (Figure 4), grouped by the daughter’s regime. Coupled cells showed the highest fidelity ($\sqrt{\mathrm{JSD}}\;=\;0.173\;\pm \;0.073$, n = 37), independent cells intermediate (0.221 ± 0.132, n = 27), and information-closed cells the lowest (0.276 ± 0.161, n = 26). A Kruskal–Wallis test confirmed a significant difference across regimes (H = 5.93, p = 0.051); the effect was driven by the coupled vs. information-closed contrast (Mann–Whitney U = 304, $p_{\mathrm{corr}}\;=\;0.041$, r = 0.37), while coupled vs. independent and information-closed vs. independent did not reach significance after Bonferroni correction.

**Figure 7:**
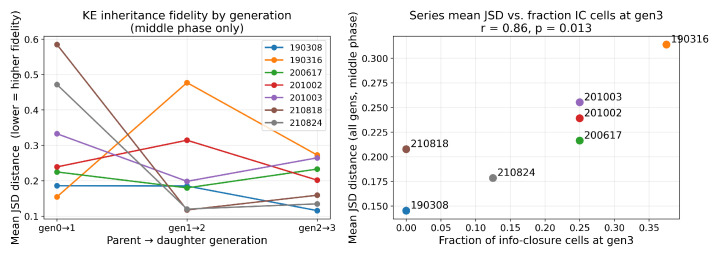
Inheritance fidelity across generation transitions (middle phase only). (**left**) Mean $\sqrt{\mathrm{JSD}}$ between parent and daughter KE distributions for each of the seven cell series across the gen0→1, gen1→2, and gen2→3 transitions. Two contrasting trajectories are visible: series 210818 and 210824 show extreme early divergence at gen0→1 followed by rapid convergence, while series 190316 shows low initial divergence and a sharp spike at gen1→2; series 190308 converges monotonically throughout. (**right**) Series-level mean $\sqrt{\mathrm{JSD}}$ (averaged over all generation transitions) plotted against the fraction of information-closed cells at gen3, used here as a proxy for the degree of categorical differentiation the community has undergone (partitioning into coupled, IC, and independent roles). The positive trend (r = 0.58, p = 0.17) suggests that communities exhibiting greater categorical differentiation also tend to show greater phenotypic divergence across generations—two complementary signatures of community-generated individuality—though the association is underpowered at n = 7 series.

Critically, the lower fidelity of information-closed cells cannot be attributed to a generic loss of coupling: independent cells, which also show low $MI(X^{\prime };C)$, do not inherit significantly worse than coupled cells. It is specifically the information-closed state—where coupling to siblings persists ($MI(X^{\prime };C)>0$) but NTIC collapses to zero—that predicts the greatest divergence from the parental KE phenotype, a direct dynamical signature of situated autonomy. The per-series breakdown and generation-level trajectory of this divergence are shown in Figure 7.

### 3.8. Per-Series Informational Diversity

The per-series breakdown reveals that each experimental community develops its own characteristic informational signature (Table 2).

Series 190316 and 201002 are information-closure-dominant, with 43% of middle-phase cells reaching the information-closed regime, and these series also show the greatest inheritance divergence (mean $\sqrt{\mathrm{JSD}}\;=\;0.38$ and 0.22 respectively for information-closed cells). By contrast, series 200617 is independent-dominant (57% independent cells), with almost no information-closed cells (7%), and exhibits a qualitatively different fidelity pattern: here it is the *independent* cells—not information-closed ones—that show elevated inheritance divergence ($\sqrt{\mathrm{JSD}}\;=\;0.23$), suggesting that in this community decoupling from collective context itself drives phenotypic divergence rather than the emergence of situated autonomy. Series 210818 and 190308 are coupled-dominant (57% and 50% respectively) and show the lowest inheritance divergence overall.

#### Inheritance Fidelity Across Generations

To examine how inheritance fidelity evolves across successive divisions, we computed the mean $\sqrt{\mathrm{JSD}}$ between parent and daughter KE distributions separately for each generation transition, restricted to the middle phase (Figure 7). At the population level, fidelity increases across generations: pooled mean $\sqrt{\mathrm{JSD}}$ declines from 0.313 ± 0.162 at gen0→1, to 0.227 ± 0.145 at gen1→2, and to 0.197 ± 0.105 at gen2→3, suggesting that the community progressively stabilizes its phenotypic structure as it matures.

Between-series trajectories reveal two contrasting patterns (Figure 7 left). Series 210818 and 210824 show extreme early divergence at gen0→1 ($\sqrt{\mathrm{JSD}}\approx 0.59$ and 0.47 respectively), followed by rapid convergence in subsequent generations; the founder cell appears to carry a distinctive phenotype that scatters widely in the first division, after which daughters progressively re-synchronize. Series 190316 exhibits the opposite trajectory: low initial divergence at gen0→1 (0.15), a sharp spike at gen1→2 (0.48), and partial recovery thereafter, suggesting that phenotypic diversity erupts in the second generation rather than the first. Series 190308 follows a monotonically convergent trajectory (0.19→0.19→0.12) throughout.

Across the seven series, the fraction of information-closed cells at gen3 shows a positive upward tendency with mean inheritance divergence (Pearson r = 0.58, p = 0.17; Figure 7 right). This association is consistent with Community First Theory, but the nature of the diversity requires clarification. The community does not merely produce IC cells: it bifurcates into a mixture of coupled, information-closed, and independent cells. The IC fraction is therefore a proxy for the *degree of categorical differentiation* the community has undergone—how far it has partitioned into distinct informational roles. The JSD divergence, by contrast, measures *phenotypic* diversity: how much daughters differ from parents in their KE distributions. The positive trend suggests that communities which undergo greater categorical differentiation (more IC cells emerging alongside coupled and independent ones) also tend to exhibit greater phenotypic divergence across generations. These are two complementary signatures of community-generated individuality, and both are predicted by Community First Theory. The trend does not reach conventional significance, which is expected given there are only seven independent series; the direction and magnitude (r = 0.58) motivate follow-up experiments with larger cohorts.

### 3.9. Interpretation: Information Closure as the Relocation of Agency to the Individual Cell

#### NTIC as Redundancy Between Self-Prediction and Sibling Influence

A complementary interpretation of NTIC follows from relating it to transfer entropy from the sibling context *C* to the focal cell *X* [28]. Transfer entropy is defined as

(3) $$\mathrm{TE}\left(C\to X\right)\;=\;I\left(X^{\prime };\;C|X\right),$$

quantifying the information that the sibling configuration provides about the future state $X^{\prime }$ beyond what is already contained in the cell’s own past *X*.

Applying the chain rule for mutual information, NTIC decomposes as

(4) $$\mathrm{NTIC}\;=\;I\left(X^{\prime };\;X\right)-I\left(X^{\prime };\;X|C\right)\;=\;I\left(X^{\prime };\;C\right)-I\left(X^{\prime };\;C|X\right)\;=\;I\left(X^{\prime };\;C\right)-\mathrm{TE}\left(C\to X\right).$$

Thus, NTIC measures the *redundancy* between two predictive channels: the self-predictive channel $X\to X^{\prime }$ and the sibling channel $C\to X^{\prime }$. It captures the portion of sibling information about $X^{\prime }$ that overlaps with what the cell already encodes in its own history.

This decomposition yields a clean interpretation of each regime:

1. *Coupled* (NTIC≫0): Predictive information from *C* is largely redundant with self-prediction; the individual’s predictive structure is aligned with that of the collective.
2. *Information closure* (NTIC = 0): Redundancy vanishes and $\mathrm{TE}\left(C\to X\right)\;=\;I\left(X^{\prime };\;C\right)$, meaning that sibling information reaches the cell’s future through a channel that does not overlap with self-prediction—the collective contributes, but in a way that is complementary to, rather than redundant with, the cell’s own history. The two predictive channels operate along informationally independent dimensions of the future state.
3. *Synergistic* (NTIC<0): Conditioning on *X* reveals additional predictive information from *C*, corresponding to genuinely relational prediction. No statistically robust cases of this regime were identified in the present dataset.

A critical subtlety for the notion of agency is that information closure does *not* imply that TE(C→X) = 0. Even when NTIC = 0, sibling influence can remain positive, so the cell is not causally isolated from its environment. Rather, closure means that the cell’s intrinsic predictive structure is undisturbed by that influence:

(5) $$I\left(X^{\prime };\;X|C\right)\;=\;I\left(X^{\prime };\;X\right).$$

This is precisely the condition for *situated autonomy*: an agent is embedded in and receives information from its surroundings (TE>0), yet its core self-dynamics remain informationally self-sufficient. By contrast, TE = 0 would represent complete causal isolation—the absence of agent–environment interaction, not its mature form. Information closure therefore marks the point at which predictive agency is relocated from the collective to the individual cell, while the cell remains an embedded member of the collective.

## 4. Discussion

### 4.1. Community First Theory: Individuality Emerges from Collective Organization

The results reported here provide initial quantitative empirical support for Community First Theory, which holds that individuality is not a pre-given property of isolated components, but an emergent informational phenomenon generated by collective organization. The central claim of the theory is that forming a community *regenerates* individuality at a new dynamical level: it is precisely through the process of collective interaction that distinct individual identities arise. The *Tetrahymena* data instantiate this claim concretely. In the early phase, when the population is small and cells have only recently divided, 97% of cells are strongly coupled—the collective dominates and individual predictive structure is largely redundant with sibling context. By the middle phase, as the eight-cell community consolidates, 27% of cells enter the information-closed regime: they remain embedded in a correlated sibling context ($I(X^{\prime };\;C)>0$) while their future dynamics become effectively self-determined (NTIC≈0). Coupling partially re-emerges in the last phase before division as the community begins to dissolve in preparation for the next division, suggesting that the differentiation of individual identities is transient and non-monotonic rather than a one-way transition.

This trajectory is not a gradual weakening of collective cohesion. It is a redistribution of predictive structure: the community generates the conditions under which distinct individual dynamical identities can crystallize. The sibling mean-field variable $Z_{t}$ in our framework is precisely the kind of collectively computed macroscopic quantity that Flack [29] identifies as the locus of downward causation: components coarse-grain the collective state and adjust their behavior accordingly. The transition from coupled to information-closed cells can therefore be read as the passage from a regime in which the macro level causally dominates the micro (high effective information at the collective scale, in the sense of Hoel et al. [30]) to one in which individual-level causal structure re-emerges. Unlike approaches that infer agency from task performance or predefined roles, NTIC provides an explicit information-theoretic criterion for locating this transition—identifying *where* predictive structure is borne, absorbed, or relocated as the collective develops.

### 4.2. Information Closure as Constructed Individual Identity

The inheritance analysis deepens this interpretation. Cells in the information-closed regime showed the greatest divergence from their parental KE attractor ($\sqrt{\mathrm{JSD}}\;=\;0.276\;\pm \;0.161$), significantly larger than coupled cells (0.173 ± 0.073; Mann–Whitney $p_{\mathrm{corr}}\;=\;0.041$, r = 0.37), and this effect was specific to the information-closed state: independent cells, which also show low $MI(X^{\prime };\;C)$, did not inherit significantly worse than coupled cells ($p_{\mathrm{corr}}\;=\;1.00$).

This specificity is theoretically important. A generic loss of coupling (the independent regime) does not destroy inheritance fidelity. What destroys it is the information-closed state, in which $MI(X^{\prime };\;C)>0$ persists but NTIC≈0. Coupled cells are pulled toward the collective attractor, which closely resembles the parental one, and so they inherit faithfully. Information-closed cells, by contrast, have constructed their own self-determined attractor—one that is no longer anchored to the inherited template. In this sense, information closure marks a dynamical bifurcation: the cell remains a member of the collective while simultaneously departing from the lineage attractor. This corresponds closely to Di Paolo’s [31] notion of adaptive autonomy—rooted in the autopoietic tradition [32]—where an agent maintains its self-organizing dynamics while remaining coupled to its environment, with the capacity to modulate that coupling. This is precisely what Community First Theory predicts. The community does not merely reorganize existing individual identities; it generates new ones, and these new identities are recognizable precisely by their departure from what was inherited.

### 4.3. Relation to the Classical Definition of Information Closure

The definition of information closure used here differs in an important respect from the classical formulation. In the original systems-theoretic treatment [16], closure is defined as the condition

(6) $$\mathrm{TE}\left(C\to X\right)\;=\;I\left(X^{\prime };\;C|X\right)\;=\;0,$$

meaning that the collective context provides *no* additional information about the cell’s future once its own past is known. Under this strict definition, the cell is causally isolated from its environment: it neither uses nor is influenced by sibling dynamics.

We argue that this classical condition is *neither sufficient nor necessary* for situated autonomy, and that it conflates three distinct phenomena. The condition $\mathrm{TE}(C\to X^{\prime })\;=\;0$ is equivalent to $MI(X^{\prime };\;C)\;=\;\mathrm{NTIC}$, which encompasses: (i) independence, where $MI(X^{\prime };\;C)\;=\;\mathrm{NTIC}\;=\;0$ (no collective relationship); (ii) coupling, where $MI(X^{\prime };\;C)\;=\;\mathrm{NTIC}>0$ (collective relationship dominates self-prediction); and (iii) information closure, where $MI(X^{\prime };\;C)>0$ but NTIC≈0 (collective relationship exists but does not compromise autonomy). Only the third regime—which violates the classical condition—constitutes genuine situated autonomy.

In other words, TE = 0 achieves closure trivially, through isolation. NTIC≈0 achieves closure non-trivially, through autonomy within coupling. A genuine agent is not causally sealed off from its environment; it maintains an autonomous self-model while remaining situated within an informative collective context. The *Tetrahymena* data support exactly this picture: the information-closed cells identified here have positive TE—they do receive sibling influence—yet their self-dynamics are informationally self-sufficient.

This non-trivial closure is the empirical basis of Community First Theory. The condition NTIC≈0 with $MI(X^{\prime };\;C)>0$ means that the individual’s future is not written inside the individual alone—it is sustained by the relational structure of the collective. Agency, in this sense, does not reside within the component but is granted to it through collective organization. A collective forms first, and from that collective a new form of individual autonomy emerges.

### 4.4. Informational Diversity Without Genetic Difference

The per-series results (Table 2, Section 3.8) demonstrate that the *type* of informational differentiation a community undergoes—whether cells partition into coupled vs. information-closed states, or into coupled vs. independent states—has measurable consequences for how faithfully motility phenotypes are transmitted across generations. This between-series variability is a key prediction of Community First Theory: since individuality is constructed through collective interaction rather than read off from a genetic template, the specific identities that emerge depend on the history and dynamics of each particular community. What might be called “personality” in a clonal population—the dynamical and informational differentiation of cells sharing identical genomes—is therefore a direct expression of community-generated individuality, not a genetic artifact. This finding bears on the concept of organismality [33] and the broader question of what constitutes a biological individual [34]: our clonal *Tetrahymena* population is genetically uniform yet exhibits non-trivial conflicts of informational interest, placing it at an intermediate point on the society–organism continuum. This resonates with the isologous-diversification theory of Kaneko and Yomo [35], which showed theoretically that interacting identical cells can spontaneously differentiate through dynamical instabilities; our information-theoretic classification provides empirical evidence that such differentiation manifests as distinct informational regimes. From a systems-theoretic perspective, this coexistence of informational role differentiation within a coupled collective parallels Tononi et al.’s [36] notion of neural complexity, where high complexity arises from the simultaneous presence of functional segregation and functional integration among homogeneous units, and resonates with Integrated Information Theory’s emphasis on irreducible causal structure [37]. The generation-level trajectory (Section 3.8) further shows that the timing of phenotypic diversification itself varies across communities, with some series diverging sharply at the first division and others not until the second, suggesting that the collective interaction history shapes both the degree and the tempo of individuation.

### 4.5. Connection to Partial Information Decomposition

The identity NTIC = Rdn−Syn (Equation (2)) places our framework within the partial information decomposition (PID) literature [18], where the total mutual information $MI(X_{t},\;C_{t};\;\;X_{t+1})$ is decomposed into redundant, unique, and synergistic components. The specific values of PID components depend on which redundancy measure is adopted, and different PID frameworks yield different decompositions. However, adopting the minimal mutual information as redundancy [19,38],

(7) $$\mathrm{Rdn}\;=\;\min\left\{MI\left(X_{t};\;X_{t+1}\right),\;MI\left(C_{t};\;X_{t+1}\right)\right\},$$

leads to a particularly clean consequence for information closure. If the empirical condition

(8) $$MI\left(X_{t};\;X_{t+1}\right)>MI\left(C_{t};\;X_{t+1}\right)$$

holds—meaning a cell predicts its own future better from its own past than from its siblings’ current state—then $\mathrm{Rdn}\;=\;MI(C_{t};\;X_{t+1})$. Substituting into NTIC = Rdn−Syn, information closure (NTIC≈0) then implies

(9) $$\mathrm{Syn}\approx \mathrm{Rdn}\;=\;MI(C_{t};\;X_{t+1})>0.$$

In other words, under this redundancy measure and condition (8), information closure *guarantees* that genuine synergy is present: the cell’s past and the sibling context jointly predict the cell’s future in a way that neither does alone. The collective does not merely correlate with the cell; it actively participates in constructing the cell’s future predictability.

We verified condition (8) empirically across all seven series in the middle phase. $MI(X_{t};\;X_{t+1})$ exceeded $MI(C_{t};\;X_{t+1})$ for all 98 cells (100%), with mean values of 0.940 ± 0.365 bits and 0.050 ± 0.054 bits respectively—a nearly 19-fold difference confirming that temporal self-prediction dominates sibling-to-future coupling throughout. Consequently, ${\mathrm{Rdn}}_{\min}\;=\;MI\left(C_{t};\;X_{t+1}\right)$ holds universally in this dataset.

Because NTIC = Rdn−Syn holds cell by cell, the three informational regimes map directly onto qualitatively distinct PID signatures without requiring separate estimation of Rdn and Syn:

- Coupled (NTIC≫0): Rdn>Syn—the cell’s past and the community context carry overlapping predictive information.
- Information-closed (NTIC≈0 with MI(X;C)>0): Rdn≈Syn—the algebraic consequence of NTIC = 0 *uniquely* characterizes this regime. Both *X* and *C* contribute to predicting $X^{\prime }$, but their contributions are complementary: redundancy is near zero, so the predictive information carried by the cell’s own past and by the collective context does not overlap. Each source reaches the cell’s future through an independent channel. Because $\mathrm{Rdn}\;=\;MI(C_{t};\;X_{t+1})>0$, non-trivial synergy is present: the cell’s past and the sibling context jointly predict the cell’s future in a way that neither source does alone.
- Independent (MI(X;C)≈0): Rdn≈Syn≈0 trivially, because the cell is decoupled from its siblings.

Thus, Rdn≈Syn>0 is the specific informational fingerprint of information closure and does not arise in either the coupled or the independent case.

The adoption of minimal mutual information as redundancy is a specific assumption within the PID framework, and the quantitative values of Rdn and Syn depend on this choice. However, the qualitative conclusion—that information closure implies non-trivial synergy between self-history and collective context—is robust to this choice whenever condition (8) holds, which it does universally here.

### 4.6. Synergy and the Limits of the Present Dataset

Although a formal classification allows for a synergistic regime (NTIC<0, where relational combinations carry predictive structure not accessible from individual histories alone), the present data provide no evidence for such a regime.

In the middle phase, five cells (5.1% of 98) showed negative NTIC alongside significant MI(X;C), making them prima facie synergistic candidates. However, each cell’s significance is assessed against its own surrogate distribution (50 permutations of $Z_{t}$), giving a cell-specific lower threshold at the fifth percentile. Of the five candidates, four had NTIC values within their individual noise ranges (i.e., NTIC > cell-specific lower threshold) and were therefore classified as information-closed. Only one cell—cell 6 of experiment 201002 (generation 2, NTIC = −0.039 bits)—fell below its cell-specific lower threshold (−0.027 bits) and was formally classified as synergistic. This single case (1/98 = 1.0%) is consistent with the expected false-positive rate (α = 5%), and MI(X;C) for this cell only marginally exceeds its significance threshold, further supporting a type-I error interpretation. Notably, the overall count of five negative-NTIC cells (5.1%) matches the FPR almost exactly, reinforcing the conclusion that no genuine synergistic regime is present in this dataset.

Within Community First Theory, synergistic regimes would correspond to a pre-specialization phase in which collective roles have not yet stabilized; the present data do not support this interpretation, and we treat information closure as the primary finding.

### 4.7. Broader Implications and Future Directions

A central implication of Community First Theory is that agency relocation is not tied to a particular biological substrate, but may arise generically in interacting systems when predictive structure is redistributed between individuals and the collective context. Future work should compute NTIC and related multivariate information measures directly in robotic and multi-agent artificial systems, and examine how learning, environmental heterogeneity, and communication constraints shape the emergence of information closure. Networks of LLM-based agents offer a particularly tractable test case, as individual module histories and collective context variables are directly accessible.

The contrast with prior *Tetrahymena* studies clarifies the scope of Community First Theory. Jordan et al. [12] and Chuang et al. [13] characterized phenotypic diversity and succession dynamics at low cell densities where inter-individual interactions are sparse; in that regime, behavioral diversity is largely an intrinsic property of isolated cells. Our microchamber experiments operate in a qualitatively different, strongly interacting regime: eight cells confined to an 800 μm chamber interact continuously throughout the growth cycle, and it is precisely this strong coupling that enables the community formation we observe. The between-series heterogeneity in informational regime (Table 2) and the generation-level fidelity trajectories (Figure 7) are therefore not merely reflections of pre-existing individual variation but signatures of community-mediated differentiation inaccessible to low-density experiments. Furthermore, recent single-cell RNA sequencing of clonal *Tetrahymena* cultures has independently revealed distinct transcriptional subpopulations sharing identical genomes [14], demonstrating that community-generated individuality manifests at multiple biological levels—from gene expression to motility dynamics. Community First Theory provides the unifying explanatory framework: individuality in the strongly interacting regime is not inherited by isolated cells but emerges from, and is structured by, collective dynamics.

These findings align with recent views that agency is reorganized—rather than simply suppressed—across the unicellular-to-multicellular transition [2], and connect to information-theoretic definitions of agents based on spatiotemporal predictive structure [17]. Community First Theory provides a unifying framework for these perspectives: it is not that multicellularity suppresses cellular agency, but that collective organization generates a new level of individual identity—one that is informationally distinct from both the coupled collective and the independent isolated cell.

Several limitations should be noted. The present study examines a single biological system (*Tetrahymena thermophila*) with seven independent communities and one dynamical observable (kinetic energy), each community comprising eight cells at the third generation. Although the sample size is sufficient to establish statistical significance for the main findings, confirming the generality of Community First Theory requires extension to other interacting systems. We are currently applying related information-theoretic frameworks to honeybee colonies of order $10^{3}$ individuals [39] and to *Pristomyrmex punctatus* ant societies of order $10^{2}$ [40], where both the number of interacting individuals and the dimensionality of behavioral observables far exceed those of the present study. These ongoing analyses will test whether the coupled-to-closure transition is a general feature of collective individuation or specific to the micro-confinement geometry used here.

## 5. Conclusions

In this study, we introduced non-trivial information closure (NTIC) as an information-theoretic measure to characterize where predictive and causal structure is effectively localized in interacting systems. By analyzing complete individual-level dynamics in populations of *Tetrahymena*, we showed that predictive structure can relocate from individual temporal continuity to collective relations as interaction strength and organization increase.

In the coupled regime (NTIC≫0), a cell’s future is largely predictable from the collective context alone: the community determines the individual. In the information-closed regime (NTIC≈0 with $MI(X^{\prime };C)>0$), the cell remains embedded in the collective yet its future has become predictable from its own past—an autonomous individual has emerged. Crucially, this autonomy is not a pre-existing property; it is *constructed* through collective interaction. The community first produces individuals whose futures it controls, and from that coupled state, information closure arises: the collective gives rise to an individual that no longer requires the collective for its own prediction.

Remarkably, genetically identical populations spontaneously differentiate into distinct informational regimes—coupled, information-closed, and independent—whose proportions vary across experimental series. This categorical diversity arises without genetic difference, demonstrating that collective organization alone is sufficient to generate functional individuality.

Inheritance fidelity, measured by Jensen–Shannon divergence between parent and daughter kinetic-energy distributions, is significantly lower for information-closed cells than for coupled cells (p = 0.041), and fidelity increases across successive generations in most series. Information-closed cells depart from the inherited parental attractor, constructing new self-determined phenotypes; the community does not merely reorganize existing identities but generates new ones.

The robustness of NTIC under temporal coarse-graining further demonstrates that this measure captures a structural property of predictive organization rather than a scale-dependent artifact.

Together, these results provide empirical support for Community First Theory. The central claim is that agency does not reside inside the individual but in the relations among individuals: a collective forms first, and from that collective a new form of autonomy is granted to the individual. Within the community, each cell retains a self-predictive structure ($I(X^{\prime };\;X)>0$), and this structure persists even when collective context is accounted for ($I(X^{\prime };\;X|Z)>0$)—the individual is genuinely autonomous. Yet this autonomy is not intrinsic; it is constituted by the organizational structure of the community. The condition NTIC≈0 with $MI(X^{\prime };\;C)>0$—non-trivial information closure—is the quantitative signature of this process: the individual’s future is sustained by collective relational structure, yet has become self-determined. Individuality is not a prerequisite for collective behavior but an emergent product of it.

While a synergistic regime (NTIC < 0) was not statistically resolved in the present dataset, it may be obscured by finite-sample noise in this small-N system; we expect such a regime to become accessible in larger self-organizing collectives such as social insect colonies, multi-agent language model systems, and neural populations, where relational predictive structure can dominate over individual histories. 

## Figures and Tables

**Table 1:** Mean NTIC values across temporal coarse-graining scales and developmental phases.

Block Size	Early (Mean)	Middle (Mean)	Last (Mean)
N =1	∼0.51	∼0.03	∼0.12
N = 5	∼0.81	∼0.10	∼0.24
N = 10	∼0.91	∼0.12	∼0.28

**Table 2:** Per-series category distribution (middle phase, n = 14 per series) and mean inheritance distance $\sqrt{\mathrm{JSD}}$ for information-closed and coupled cells. IC = information-closed.

Series	Coupled	IC	Independent	Anomalous	$\sqrt{\mathrm{JSD}}$ (IC)	$\sqrt{\mathrm{JSD}}$ (Coupled)
210824	29%	21%	36%	14%	0.135 ± 0.003	0.116 ± 0.018
210818	57%	21%	14%	7%	0.486 ± 0.174	0.141 ± 0.027
190308	50%	29%	21%	0%	0.153 ± 0.075	0.154 ± 0.051
200617	21%	7%	57%	14%	0.270 ± —	0.131 ± 0.037
190316	43%	43%	14%	0%	0.377 ± 0.159	0.209 ± 0.093
201003	43%	21%	36%	0%	0.289 ± 0.161	0.243 ± 0.072
201002	21%	43%	14%	14%	0.218 ± 0.107	0.204 ± 0.124

## Data Availability

The raw data supporting the conclusions of this article will be made available by the authors on request.

## Abbreviations

The following abbreviations are used in this manuscript:

NTIC	Non-Trivial Information Closure
KE	Kinetic Energy
MI	Mutual Information
CMI	Conditional Mutual Information
JSD	Jensen–Shannon Divergence
PID	Partial Information Decomposition

## Appendix A. Statistical Significance

### The Threshold Procedure

For each individual cell in each phase:

1. Keep X(t) and X(t+1) exactly as they are—the cell’s own KE time series is untouched.
2. Shuffle C(t) randomly—permute the sibling mean KE, destroying its temporal alignment with *X* while preserving its marginal distribution.
3. Compute $MI(X;\;C_{\mathrm{shuffled}})$ and ${\mathrm{NTIC}}_{\mathrm{shuffled}}$ on this shuffled data.
4. Repeat 50 times—this gives 50 values of $MI(X;\;C_{\mathrm{shuffled}})$ and 50 values of ${\mathrm{NTIC}}_{\mathrm{shuffled}}$ for that specific cell.
5. Take the 95th percentile of those 50 values—this is the threshold for that cell.

## Appendix B. Supplementary Methods: Device Design and Staining Procedures

### Appendix B.1. Microchamber Design Considerations

To enable continuous tracking of freely swimming *Tetrahymena* cells over an entire generation, cells were required to remain within the microscopic field of view and within the focal plane. For this purpose, the chamber depth was set to approximately 15 μm, restricting motion primarily to a quasi-two-dimensional layer while preserving natural swimming behavior.

The chamber diameter was chosen as 800 μm (approximately 20 times the cell body length, ∼50 μm). At this spatial scale, cells explored both the chamber interior and boundary regions. In preliminary experiments, chambers with smaller diameters induced persistent wall-following behavior, such that cells predominantly swam along the inner boundary rather than traversing the interior. This reduced the diversity of observable trajectories and limited opportunities for cell–cell interactions. The selected chamber geometry therefore provided a balance between confinement for complete tracking and sufficient free space for natural motility.

A sheet-type PDMS device containing nine such cylindrical chambers arranged in a 3×3 array was fabricated and used throughout the experiments (Figure 1b).

### Appendix B.2. Ink Staining Protocol for Stereomicroscopy

For two stereomicroscopic recordings (datasets 210818 and 210824), cells were stained to enhance contrast for reliable tracking. Cells were mixed 1:1 (*v*/*v*) with a 1% (*v*/*v*) suspension of Dr. Ph. Martin’s^®^ BLACK STAR^®^ waterproof india ink (MATTE; Salis International, Inc., Golden, CO, USA) and incubated at room temperature for approximately 2 h. After incubation, stained cells were diluted in fresh PPY medium prior to loading into the PDMS microchamber device. No qualitative differences in proliferation or gross motility were observed under these staining conditions during the recording period.

### Appendix B.3. Long-Term Stabilization and Evaporation Suppression

To permit sustained observation over extended durations (up to ∼14 h), chambers were sealed with culture inserts equipped with porous PET membranes (0.4 μm pore size). These membranes prevented cell escape while allowing diffusion of oxygen and nutrients (Figure 1c).

To suppress evaporation and maintain stable medium composition, a layer of silicone oil (AR200; Sigma-Aldrich, Burlington, MA, USA) was applied above the culture medium reservoir. In the stereomicroscopic configuration, mechanical stabilization was achieved by pressing the inverted PDMS device against the membrane insert using a weighted glass slide. This assembly formed a confined yet physiologically stable environment for long-term imaging (Figure 1c).

All datasets and chamber geometries were identical across experiments unless otherwise noted.
